# BMSC-derived exosomes from congenital polydactyly tissue alleviate osteoarthritis by promoting chondrocyte proliferation

**DOI:** 10.1038/s41420-020-00374-z

**Published:** 2020-12-10

**Authors:** Xinghua Zhou, Hansi Liang, Xiaohan Hu, JinNan An, Sisi Ding, Shuichang Yu, Cuiping Liu, Fang Li, Yunyun Xu

**Affiliations:** 1 Institute of Pediatrics, Children’s Hospital of Soochow University, Suzhou, Jiangsu China; 2 Department of Human Anatomy, Histology and Embryology, School of Biology and Basic Medical Sciences, Soochow University, Suzhou, Jiangsu China; 3 Jiangsu Institute of Clinical Immunology, The First Affiliated Hospital of Soochow University, Suzhou, Jiangsu China; 4 Institute of Blood and Marrow Transplantation, The First Affiliated Hospital of Soochow University, Suzhou, Jiangsu China

**Keywords:** Mesenchymal stem cells, Stem-cell differentiation

## Abstract

In the past decade, mesenchymal stem cells (MSCs) have been widely used for the treatment of osteoarthritis (OA), and exosomes may play a major role. Here, we acquired a special kind of MSCs from the bone marrow of surgically resected tissue from the hand of a patient with polydactyly. Experiments were focused on the role of polydactyly bone marrow-derived MSCs (pBMSCs) in osteoarthritis. The results showed that the pBMSCs had a greater ability than the BMSCs to differentiate into chondrocytes. Mechanistically, the expression of BMP4 was significantly higher in the pBMSCs than it was in the BMSCs. Furthermore, we showed that the migration and proliferation of chondrocytes were stimulated by exosomes secreted by pBMSC (pBMSC-EXOs). Notably, the downregulation of BMP4 in pBMSCs by siRNA inhibited both the chondrogenic differentiation potential of the MSCs and the function of the chondrocytes. In addition, the injection of pBMSC-EXOs and BMSC-EXOs attenuated OA in an OA mouse model, but the pBMSC-EXOs had a superior therapeutic effect compared with that of the BMSC-EXOs. Taken together, the data indicate that pBMSCs have greater ability to differentiate into chondrocytes and regulate chondrocyte formation through BMP4 signaling. Therefore, pBMSC-EXOs may represent a novel treatment for OA.

## Introduction

Osteoarthritis (OA) is a chronic joint disease characterized by degeneration, destruction, and osteogenesis of joint cartilage. It is a “total joint disease”; that is, all the tissues in the joint are involved in the pathogenesis of OA^1,2^. The incidence of OA is closely related to the advancing age, and the prevalence rate of OA in females is significantly higher than that it is in males^3^. The pathogenesis of OA is complex and has a heritable tendency^4^. In general, it is believed that, due to the interaction of mechanical and biological factors, imbalances in articular chondrocytes, the extracellular matrix (ECM) and the extent of subchondral bone synthesis and degradation are the main reasons for the development of OA^5^. Currently, nondrug therapy, drug therapy, and surgical treatment in the clinic do not fundamentally delay the progressive degeneration of articular cartilage during OA. In view of this, a new cell-based treatment of OA has attracted increasing attention.

Mesenchymal stem cells (MSCs), with the potential of self-renewal and directed differentiation, can repair cartilage tissue and inhibit the secretion of inflammatory factors by chondrocytes. Furthermore, MSCs can be directed to differentiate into chondrocytes in vivo, which provides a theoretical basis for the treatment of OA by MSCs. Moreover, a series of studies confirmed that MSCs can effectively treat OA from different aspects^6–8^. Clinical application has preliminarily proven that MSCs may be the best method to treat traumatic bone and cartilage defects^9^. However, the therapeutic effects of BMSCs are not ideal^10^. In addition, the risk of tumor formation, ethical issues, and transplant rejection remain barriers to further stem cell studies^11^. Besides, unified standards for the isolation methods of stem cells and the optimal storage conditions needed to maintain cell viability are lacking, which severely limits the further clinical application of stem cell therapy. Consequently, new strategies to overcome these disadvantages are still needed.

In recent years, research on the paracrine effect of stem cells in the process of tissue repair has attracted considerable attention. Exosomes are tiny vesicles released into the ECM upon the fusion of multivesicular bodies with the cytoplasmic side of the plasma membrane. Exosomes are shaped like discs and have a diameter of 30–150 nm^12–14^. In addition, exosomes contain a variety of proteins, lipids, and nucleic acids with important functions and can transmit these specific components as signal molecules to both nearby and distant cells, affecting the function of recipient cells. In recent years, exosomes from stem cells have been shown to be involved in the regeneration of myocardial tissue^15,16^, lung tissue^17,18^, the retina^19^, central nervous system^20^, kidney tissue^21^, and pancreatic tissue^22^. Our previous studies of amniotic fluid stem cell (AFS) transplantation for skin injury repair have suggested that transplanted human AFS does not promote tissue repair by differentiating into the same type of cells in damaged tissue but by affecting the wound repair microenvironment through paracrine signaling that promotes the repair of damaged skin tissue^23^, which is additional evidence showing that stem cell-derived exosomes may be an alternative to cell therapy.

Congenital polydactyly is manifested as a malformation of the hand, and surgical resection is currently the common treatment for this disease^24^. With informed consent, we obtained a special type of mesenchymal stem cells (pBMSCs) from bone marrow extracted from the soft tissue of surgically resected fingers. Subsequently, using immunophenotypic identification and functional analysis, we found that pBMSCs have greater capacity than BMSCs for in vitro amplification. Importantly, pBMSCs have a profound ability to differentiate into chondrocytes. In addition, the BMP4 pathway plays not only a regulatory role in the differentiation of pBMSCs into chondrocytes but also an important role in the promotion of the growth of chondrocytes through the secretion of pBMSC-derived exosomes (pBMSC-EXOs). Finally, pBMSC-EXOs injection into the joint cavity significantly improved cartilage injury in the knee of a collagenase-induced OA mouse model, as shown by the OARSI score. Here, our study suggests that pBMSC-EXOs, once considered “medical waste”, can function as a treatment for OA when administered properly.

## Materials and methods

### Primary cell cultures

#### pBMSCs and BMSCs

Polydactyly tissue was obtained by surgical resection during treatment for congenital polydactyly disease. pBMSCs were collected from bone marrow aspirates of this polydactyly tissue, while BMSCs were collected from bone marrow aspirates of healthy adult donors. Written informed consent was obtained from all patients and donors. The method for BMSCs preparation was described previously^25^. Moreover, the collected cells were cultured in low-glucose Dulbecco’s modified Eagle’s medium (LG-DMEM) supplemented with 10% fetal bovine serum (FBS; Gibco, CA, USA).

#### Chondrocytes

The articular cartilage of limbs from the polydactyly tissue sample was separated under aseptic conditions, and then, the fascia and cartilage membrane surrounding the cartilage tissue were peeled off. Next, the cartilage tissue was cut into ~0.5-mm tissue blocks and washed three times with PBS containing penicillin and streptomycin. The cartilage tissue was digested for 1–2 h with 0.25% trypsin at 37 °C. After trypsin digestion, the cartilage tissue continued to be digested overnight with 0.02% type II collagenase at 37 °C, and finally, a single cell was isolated. The isolated chondrocytes were collected and cultured with DMEM/F12 containing 10% FBS.

All the cells used were analyzed between passage 3 and 5. The use of the pBMSCs, BMSCs, and chondrocytes was approved by the Ethics Committee of Soochow University (approval No. SUDA20200707H01).

### Characterization of BMSCs and pBMSCs

The immunophenotypes of the BMSCs and pBMSCs were determined by flow cytometry analysis. The cells were harvested and incubated with monoclonal antibodies against CD105, CD106, CD34, CD90, CD45, CD133, CD73, CD14, and HLA-DR or the isotype control (eBioscience) for 30 min at 4 °C. Later, the surface antigens of the cells were analyzed using a Gallios flow cytometer and analyzed using Kaluza software (Beckman Coulter).

### Trilineage differentiation of the BMSCs and pBMSCs

#### Osteogenic differentiation

For osteogenic differentiation, BMSCs and pBMSCs were seeded at a density of 5 × 10^3^ cells/cm^2^. Then, MSCs were cultured with LG-DMEM containing 10% FBS, 0.1 mM dexamethasone, 50 mM vitamin C, and 10 mM glycerophosphate. After the cells grew to 60–70% confluence, the osteogenic induction medium was replaced every 3 days. After 2 weeks, the osteogenic differentiation of the BMSCs was analyzed by alizarin red staining. Mineral nodules appeared in the pBMSCs after 1 month in culture.

#### Adipogenic differentiation

For driving adipogenic differentiation, induction medium (DMEM containing 10% FBS, 1 mM dexamethasone, 200 mM indomethacin, 0.5 mM isobutylmethylxanthine and 10 mg/mL insulin) was added to the BMSCs and pBMSCs after the cells reached 100% confluence. The adipogenic induction medium was replaced every 3 days. After 2 weeks, the lipid accumulation in BMSCs was determined by oil red O staining, and the lipids appeared in the pBMSCs after 1 month.

#### Chondrogenic differentiation

For chondrogenic differentiation, the BMSCs and pBMSCs were seeded at a density of 5 × 10^5^ cells/15-mL conical tubes via centrifugation at 250 × *g* for 5 min. Then, the cells were induced with chondrogenic media (LG-DMEM containing 1% FBS, 0.01 µg/mL TGF-β3, 0.4 µg/mL dexamethasone, 0.05 µg/mL 2-phospho-l-ascorbic acid, 0.04 mg/mL proline, and 1% insulin–transferrin–selenium solution (Thermo Fisher Scientific, Waltham, MA). The induction medium was replaced every 3 days. Cell pellets were maintained in culture for 7–21 days. Finally, the pellets were identified by paraffin embedding, sectioning, and toluidine blue staining.

### Quantitative reverse transcription PCR

Total RNA was isolated using a NucleoSpin RNA kit (MACHEREY-NAGEL, Duren, Germany) and reverse transcribed with a PrimeScript Reverse Transcriptase kit (TaKaRa, Dalian, China). Next, quantitative reverse transcription PCR was performed using a HiScript II One Step qRT-PCR SYBR Green kit (Vazyme, Nanjing, China) on an iQ5 Real-time PCR Detection system (Bio-Rad, Hercules, CA, USA). Moreover, the relative gene expression was analyzed by the 2^–ΔΔCT^ method. The primers for Acan, Sox9, CoL2A1, Foxc2, BMP4, CTNNB1, c-Myc, and Actin are listed in Table S1.

### Immunofluorescence

After blocking with 5% bovine serum albumin, the BMSCs and pBMSCs were incubated overnight at 4 °C with primary antibodies: rabbit anti-human BMP4 (1:100, Abcam). Then, the cells were washed with PBS and incubated with HRP-conjugated secondary antibodies (Life Technologies, Duren, DE) for 30 min at room temperature. Finally, images were captured by fluorescence microscopy (Nikon Eclipse Ni, Tokyo, Japan) after the cell nuclei were stained with DAPI (SouthernBiotech, AL, USA).

### Isolation and identification of the BMSC-EXOs and pBMSC-EXOs

Exosomes were isolated and purified from the supernatant of the pBMSCs and BMSCs according to an established protocol^26,27^. Upon reaching 80–90% confluency, the cells were washed with PBS, and the culture medium was changed to LG-DMEM without FBS. After culturing for 48 h, the cultured supernatant was collected and centrifuged at 300 × *g* for 10 min and then at 2000 × *g* for 30 min at 4 °C. Next, the supernatant was transferred to a new tube without disturbing the pellet. Finally, the exosomes were collected according to the protocol of a total exosome isolation kit (Invitrogen^TM^, CA, USA).

The concentration and size distribution of the pBMSC-EXOs and BMSC-EXOs were measured by ZetaView (Particle Metrix, Germany). The morphology of the exosomes was determined by observation via an FEI Tecnai G2 spirit transmission electron microscope (TEM; FEI, Eindhoven, the Netherlands). Measurements of the characteristic proteins CD9 (1:1000; Abcam, Cambridge, UK), CD63 (1:1000; Abcam), and TSG101 (1:1000; Santa Cruz, Dallas, TX, USA) in the exosomes were determined by western blotting.

### Coculture experiments

The pBMSCs, BMSCs, and chondrocytes were cultured according to previous protocols. Coculture experiments were performed by seeding chondrocytes (5 × 10^4^) with pBMSCs or BMSCs (5 × 10^4^) in a six-well plate. Furthermore, chondrocytes (5 × 10^4^) were seeded in the lower chamber, and pBMSCs or BMSCs (5 × 10^4^) were seeded in the upper chamber of a six-well Transwell apparatus with a 0.4-µm pore size (Corning Incorporated, NY, USA). After 48 h, we evaluated the function of the BMSC-EXOs and pBMSC-EXOs in promoting the proliferation of chondrocytes by calculating the cell number.

### Wound-healing assay

After being treated with pBMSC-EXOs or transduced with siRNA against BMP4 and the chondrocyte culture reached 90–100% confluence, a scratch was made in the cultured monolayer. Small interfering RNA targeting specific human BMP4 sites (si480 and si1318) and a negative control siRNA (siNC) were synthesized by GenePharma (Shanghai, China) (Table S2). Then, the cells were washed with PBS to remove detached cells. Images of the wound area were captured at different time points (24, 48, and 72 h) by a microscope (Nikon Eclipse Ti, Tokyo, Japan) connected to a Nikon camera using NIS-Elements software.

### Migration assay

Transwell Permeable Support (Corning, NY, USA) was used for the migration assay of chondrocytes with or without pBMSC-EXOs. Coculture experiments were performed by seeding chondrocytes (5 × 10^4^) in the upper chamber and pBMSC-EXOs in the lower chamber of the system. After incubation for 24 h, the migrated cells were stained with crystal violet and counted.

### Collagenase-induced OA model

Six-week-old male C57BL/6J mice were anesthetized with sodium pentobarbital (1% (m/v), 8 mL/kg) and randomly distributed into four groups: control (*n* = 5), OA (*n* = 6), BMSC-EXOs treatment (*n* = 6), and pBMSC-EXOs treatment (*n* = 6). After anesthesia, collagenase VII (*Clostridium histolyticum*, Sigma-Aldrich, USA) was used to create the OA model according to the described protocol^28^. In the control group, the knee joints of the mice were injected with 10 μL saline intra-articularly through the patellar ligament. In the other groups, 10 μL of saline with collagenase was injected into the knee joints in the same way. For the therapeutic experiment, we injected saline, BMSC-EXOs, and pBMSC-EXOs into the OA, BMSC-EXOs, and pBMSC-EXOs groups, respectively, on days 7, 14, and 21. The mice were euthanized for further histology analysis on day 28. All operations were performed under protocols approved by the Ethics Committee of Soochow University and the international guidelines for animal experimentation (approval No. SUDA20200707A02).

### Histology

The mouse joints were isolated from the OA model and fixed with 10% buffered paraformaldehyde for 24 h. Before being embedded in paraffin, mouse tibias were decalcified in 10% EDTA (pH 7.4) according to a standard protocol^29^. Furthermore, the 5-μm-thick sagittal joint sections were stained with hematoxylin and eosin (H&E) and safranin O/fast green. The severity of the cartilage destruction was evaluated based on the Osteoarthritis Research Society International (OARSI) scoring system^30^.

### Statistical analysis

All statistical values were calculated by using SPSS v18 (SPSS, Chicago, IL, USA). Data are reported as the means ± standard deviation. Two groups were compared by unpaired Student’s *t* test, while more than two groups were assessed through one-way analysis of variance followed by Bonferroni test. A *p* value <0.05 was considered significant.

## Results

### Characterization of the pBMSCs

**Fig. 1:**
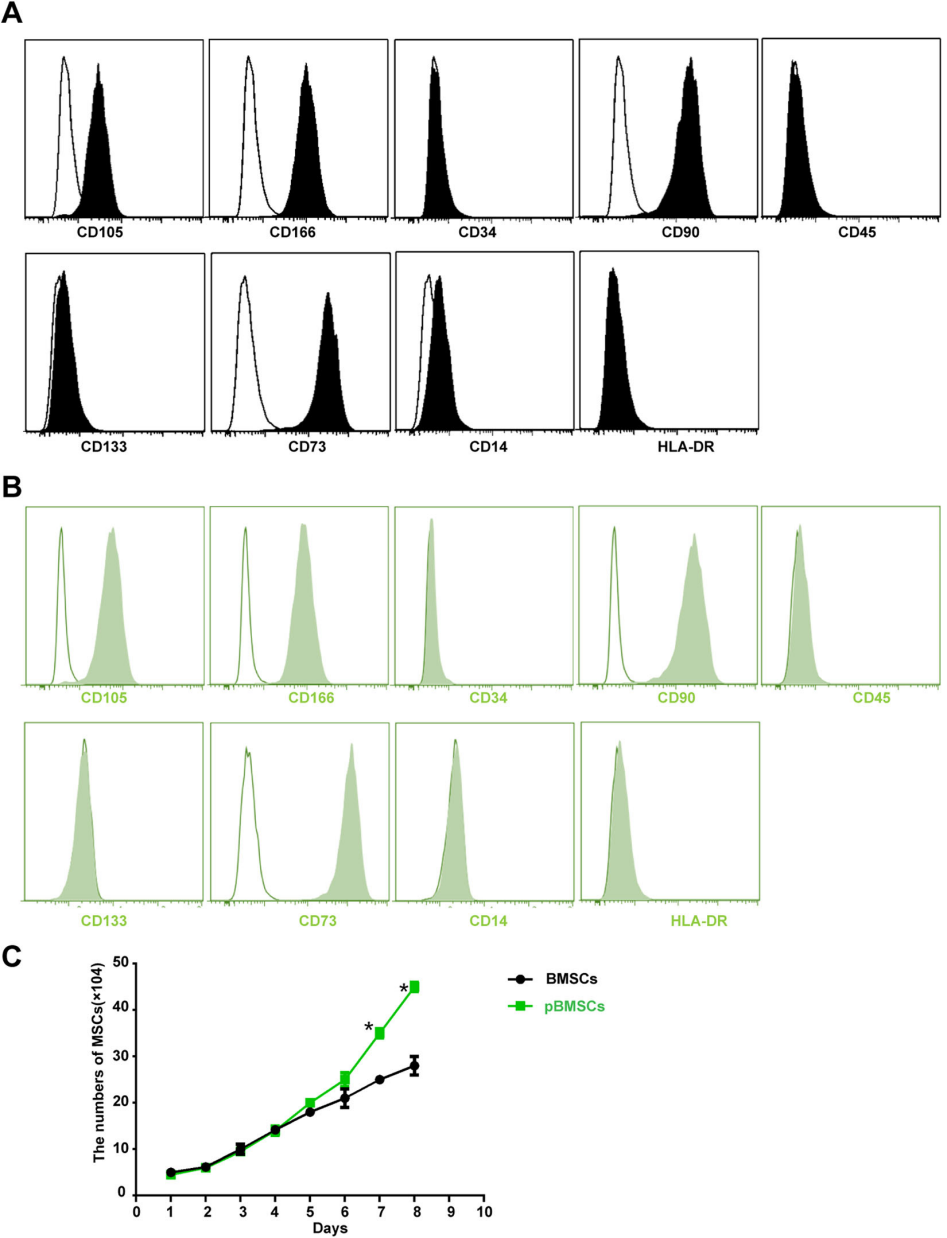
Immune phenotypes and growth curves of the pBMSCs. Flow cytometric analysis of immune phenotypes of **A** MSCs from polydactyly tissue and **B** MSCs from bone marrow. The blank areas indicate the control, and the filled areas indicate the treated cells. **C** Growth curves of the pBMSCs and BMSCs cultured in vitro, *P < 0.05.

We cultured MSCs from polydactyly tissue and then identified their specific immunophenotype. The results showed that pBMSCs had an immunophenotype similar to that of BMSCs, with high expression of MSC surface markers CD105, CD166, CD90, and CD73, no expression of hematopoietic stem cell surface markers CD133 or CD34, and no expression of immune cell surface markers CD45 or HLA-DR (Fig. 1A, B). In addition to similar immunophenotypes, we compared the biological characteristics of pBMSCs with those of BMSCs, and the results of the growth curve showed that pBMSCs had higher capacity for in vitro amplification than did BMSCs (Fig. 1C).

### Greater ability of the pBMSCs to differentiate into chondrocytes

**Fig. 2:**
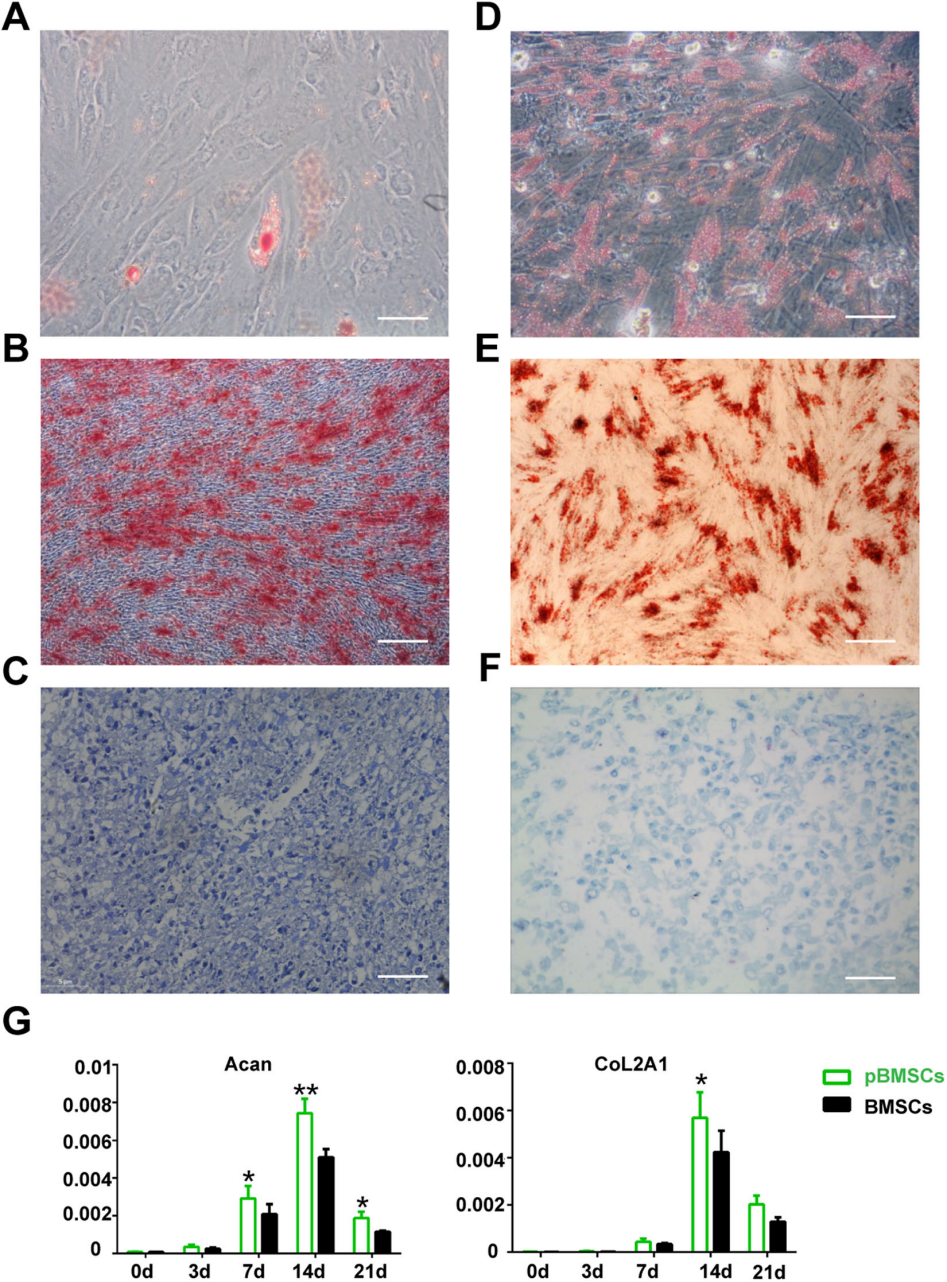
The potential of the three-dimensional differentiation of the pBMSCs. **A–C** pBMSCs were purified from polydactyly bone marrow and then induced into adipose, osteoblast, and chondroblast cells. Scale bars, 40 μm. **D–F** BMSCs were purified from donor bone marrow and then induced into adipose, osteoblast, and chondroblast cells. Scale bars, 40 μm. **G** Real-time PCR analysis of Acan and CoL2A1 mRNA expression in the pBMSCs at different time points and BMSCs after induction into chondroblast cells.

We further analyzed the ability of pBMSCs to differentiate into bone, cartilage, and adipocytes. The results showed that BMSCs differentiate into adipocytes and bone cells in 14 days and pBMSCs differentiated into adipocytes and bone cells in 60 days, which showed that pBMSCs have a lower ability than BMSCs to differentiate into adipocytes and bone cells (Fig. 2A, B, D, E). However, the differentiation time of pBMSCs into chondrocytes was 7 days, significantly less time than the 14 days for the BMSCs to differentiate into chondrocytes (Fig. 2C, F). Thus, pBMSCs have a greater ability than BMSCs to differentiate into chondrocytes. Based on this finding, we further analyzed the changes in chondrogenic genes before and after the pBMSCs and BMSCs differentiated into chondrocytes. The results showed that 14 days after induction, the expression of Acan and CoL2A1 in the chondrocytes derived from the pBMSCs was significantly higher than that in the chondrocytes derived from the BMSCs (Fig. 2G).

### pBMSCs regulate chondrocyte formation through BMP4 signaling

**Fig. 3:**
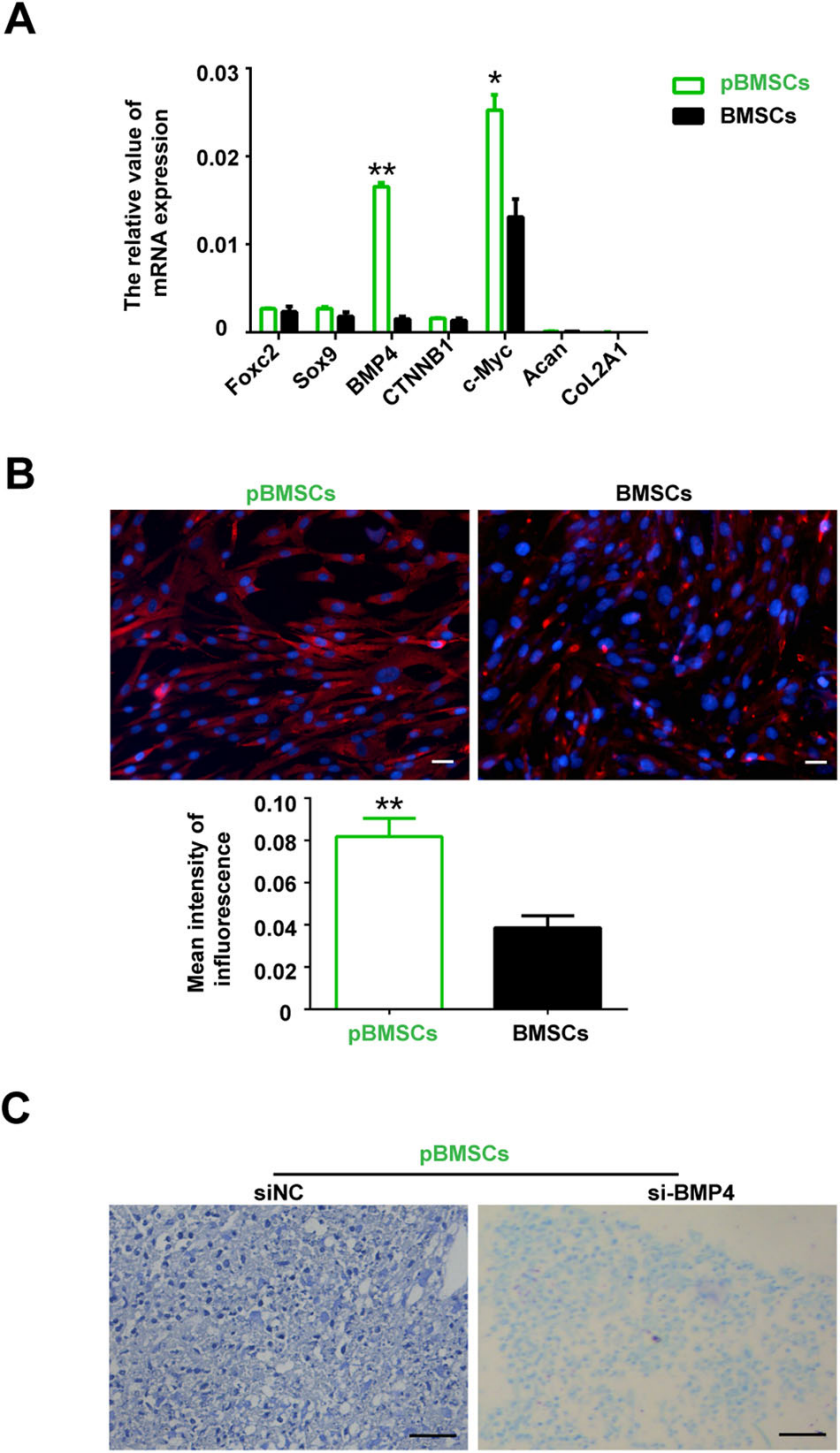
Effects of BMP4 on the pBMSCs differentiated into chondrocytes. **A** The mRNA expression of Foxc2, Sox9, BMP4, CTNNB1, c-Myc, Acan, CoL2A1, and actin was analyzed by qRT-PCR. **B** BMP4 (red) and DAPI (blue) IF staining of pBMSCs and BMSCs. Scale bars, 40 μm. **C** pBMSCs were induced to differentiate into chondrocytes after BMP4 knockdown. Scale bars, 40 μm. In addition, there was a significant difference between the BMSCs and pBMSCs groups, *P < 0.05, **P < 0.01.

In the process of chondrocyte formation, BMP4, WNT/β-catenin, and other signaling pathways play important roles. Therefore, we compared and analyzed the expression of Foxc2, Sox9, BMP4, CTNNB1, and c-myc in the pBMSCs and BMSCs. The results showed that the expression of BMP4 in the pBMSCs was significantly higher than it was in the BMSCs (Fig. 3A). In addition, the same expression pattern of BMP4 was observed at the protein level (Fig. 3B). Furthermore, after downregulation of BMP4 expression by siRNA (Fig. S1), the ability of the PBMSCs to undergo chondrogenic differentiation was inhibited (Fig. 3C).

### The exosomes secreted by the pBMSCs support the growth of chondrocytes in vitro

**Fig. 4:**
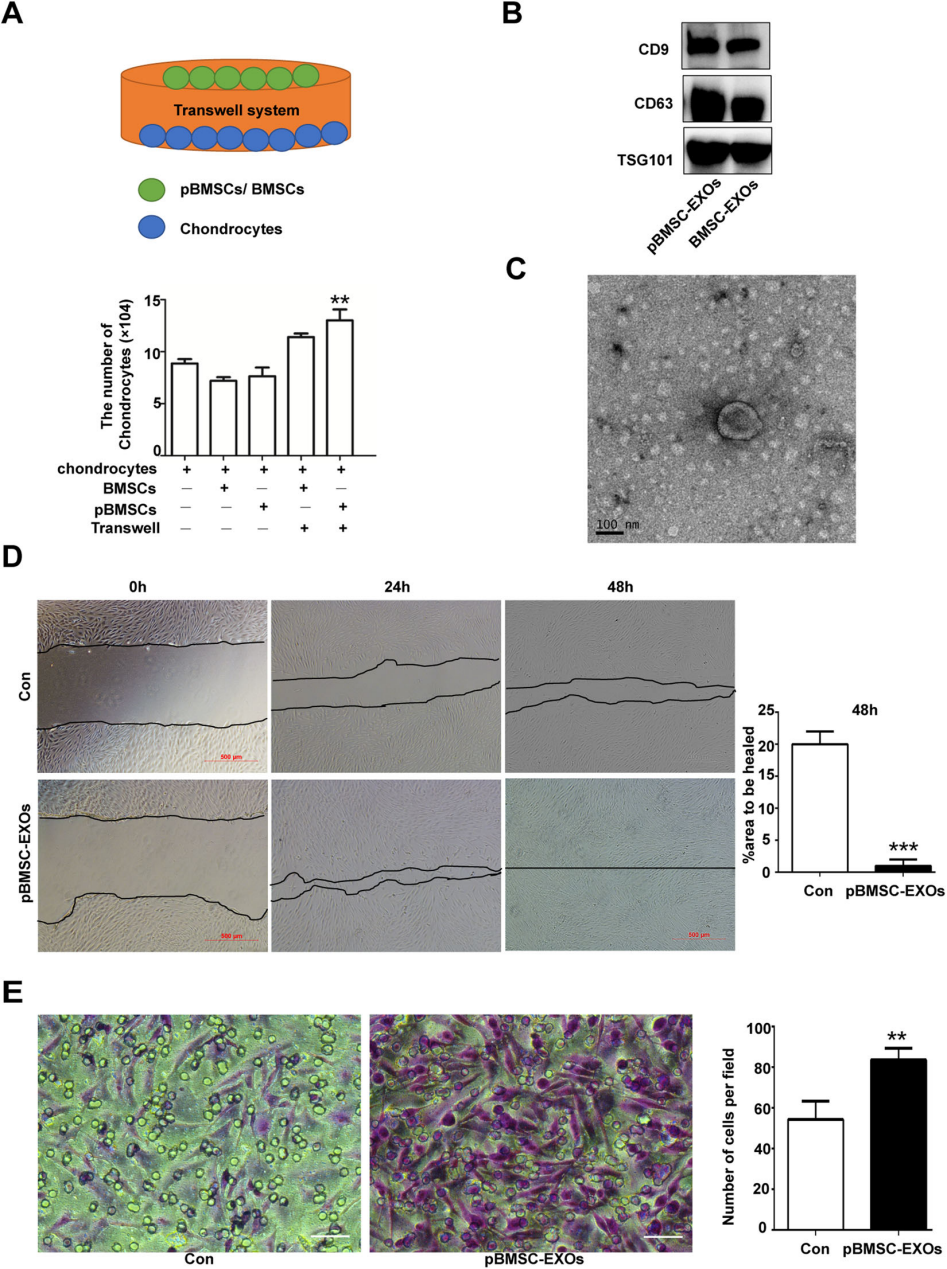
Effects of the pBMSC-EXOs on the migration and proliferation of chondrocytes. **A** The proliferation of chondrocytes in coculture with pBMSCs and BMSCs. **B** Western blot analysis of the exosome-specific CD9, CD63, and TSG101 proteins. C Morphology of the exosomes under transmission electron microscopy. Scale bars, 100 nm. **D** Wound-healing assays. **E** pBMSC-EXOs stimulated the migration of chondrocytes in the coculture system. Scale bars, 50 μm.

**Fig. 5:**
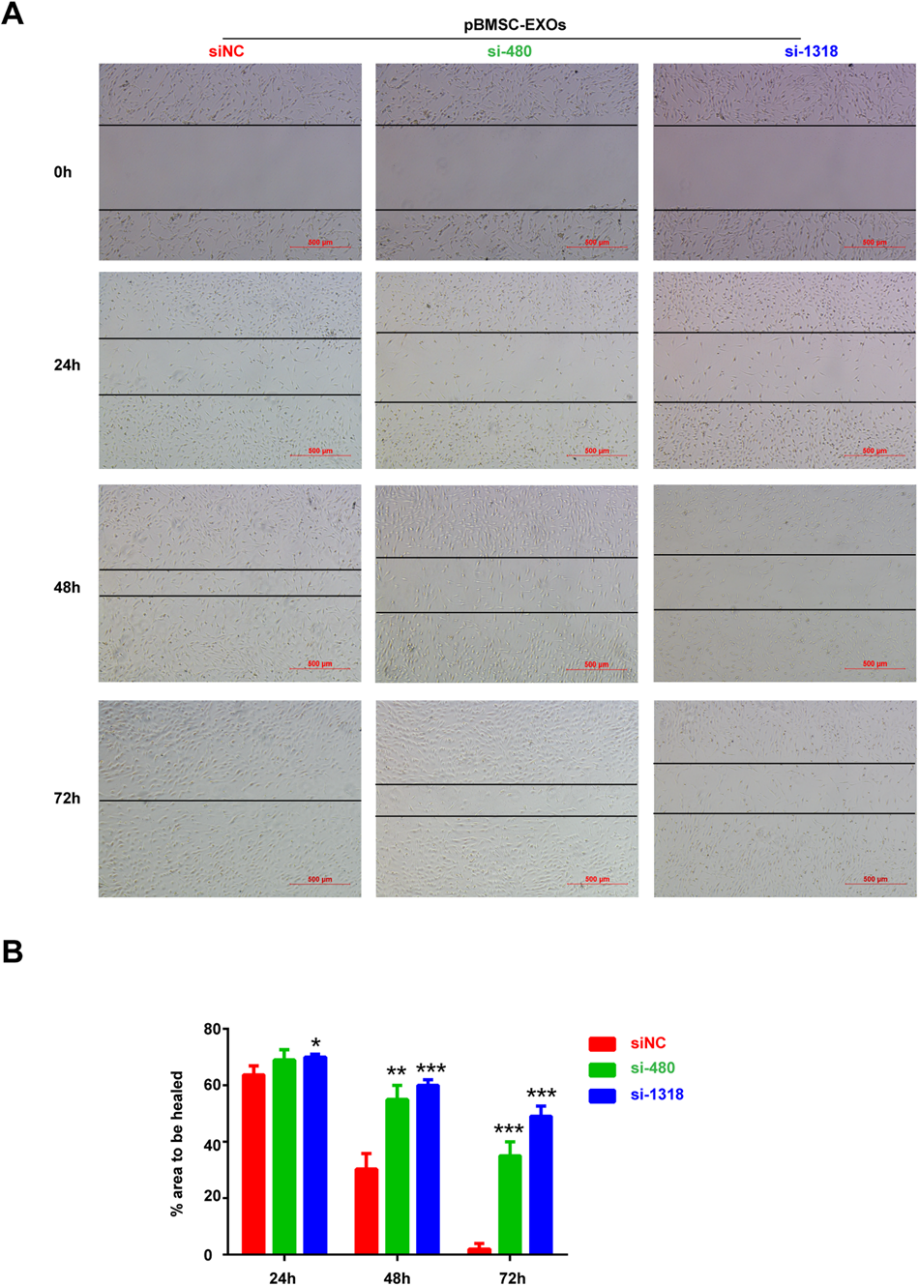
pBMSC-EXOs inhibit the migration of chondrocytes upon BMP4 knockdown. **A** Light microscopy images of wound-healing area in cultures of chondrocytes with BMP4 knocked down. Scale bars, 500 μm. **B** Quantitative analysis of the migration rates at 24, 48, and 72 h.

The pBMSCs are capable of readily differentiating into chondrocytes, but do they influence the growth of chondrocytes in vitro? To answer this question, we cocultured pBMSCs with chondrocytes to monitor the proliferation of the chondrocytes. The results showed that pBMSCs promoted chondrocyte proliferation in vitro (Fig. 4A). Compared to Microvesicles from pBMSCs, the exosomes derived from pBMSCs were mainly involved in the regulation of chondrocyte biological behaviors (Fig. S2). We isolated exosomes from the supernatant of the pBMSCs cultures by multiple overspeed centrifugation combined with a filtration method. Subsequently, we identified pBMSC-EXOs that expressed the exosome-specific proteins CD63 and CD9 (Fig. 4B). In addition, the typical 30–150 nm vesicle structure of the exosomes was observed with a transmission electron microscope (Fig. 4C). Especially, we found that pBMSC-EXOs were ingested by chondrocytes (Fig. S3), which promoted chondrocyte migration and proliferation of chondrocytes (Fig. 4D, E). Compared with pBMSC-EXOs, pBMSC-siBMP4-derived exosomes inhibited the migration and proliferation of chondrocytes, which demonstrated that BMP4 signaling also plays an important role in promoting chondrocyte proliferation (Fig. 5 and Fig. S4). In addition, pBMSC-EXOs had no effects on fibroblast-like synoviocytes (FLSs) in the proliferated synovial tissue (Figs. S5 and S6), while FLSs were involved in the destruction of articular bone and cartilage during the progression of OA.

### Exosomes secreted by the pBMSCs for the treatment of OA

**Fig. 6:**
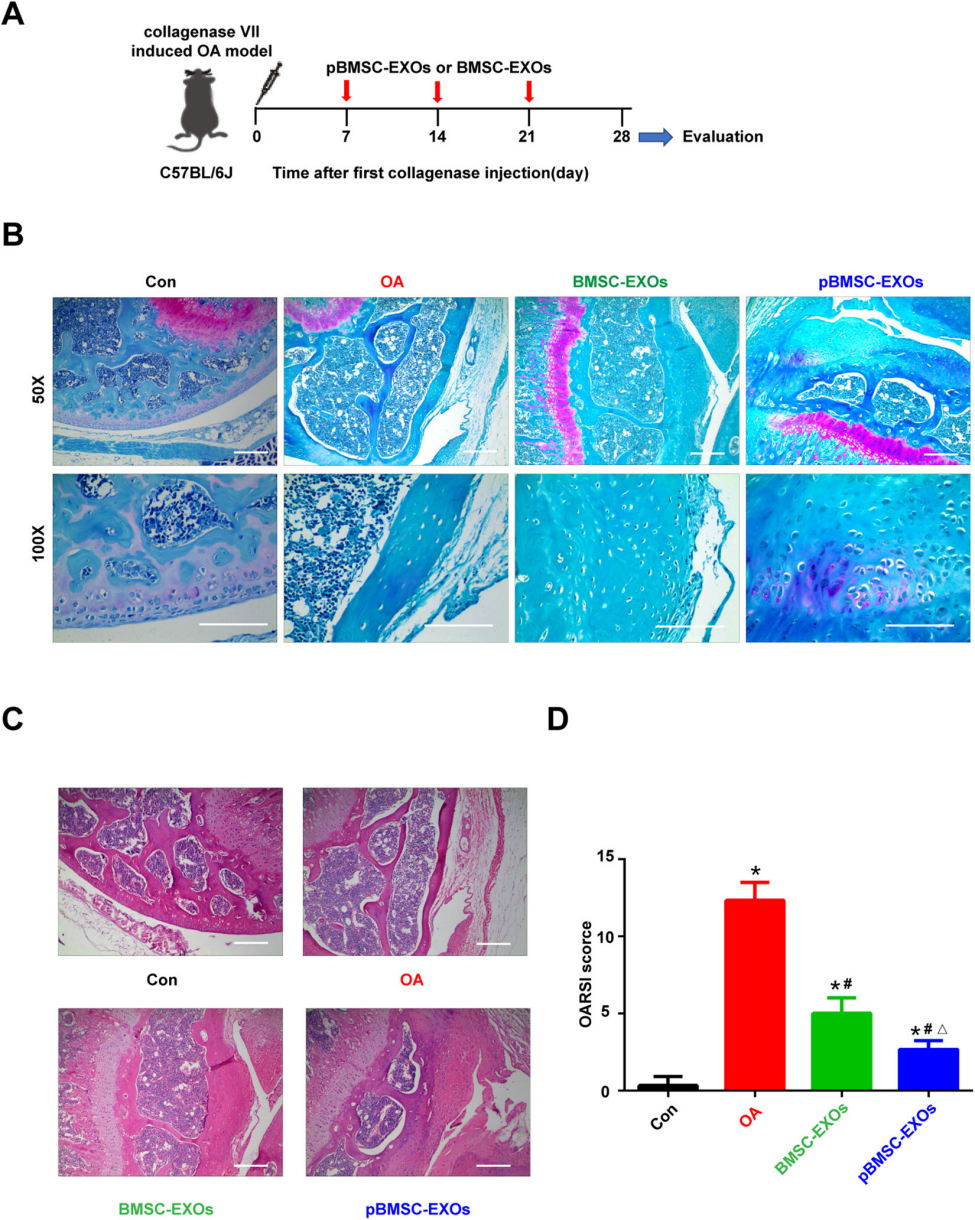
Exosomes secreted by the pBMSCs for the treatment of osteoarthritis. **A** Flowchart of the in vivo experiment. **B** Safranin O/fast green staining. Scale bars, 100 μm. **C** H&E staining. Scale bars, 100 μm. **D** OARSI scores of the OA, BMSC-EXO, and pBMSC-EXO groups were significantly higher than those of the control (Con) group, **P < 0.01. The scores of the BMSC-EXO and pBMSC-EXO groups were significantly lower than those of the OA group, ^##^*P* < 0.01. Furthermore, there was a significant difference between the BMSC-EXO and pBMSC-EXO groups, ^△^*P* < 0.05.

We constructed an OA mouse model induced by collagenase and then treated it with pBMSC-EXOs injected into the articular cavity (Fig. 6A). After 28 days of treatment, knee joint specimens (tibial plateau) were collected from the mice. The results of H&E staining and safranin O/fast green staining confirmed that pBMSC-EXOs treatment reduced the degree of cartilage damage in the OA model (Fig. 6B, C). Moreover, the OARSI scores were significantly higher for the OA model group and significantly lower for the pBMSC-EXOs treatment group than it was for the normal control group (Fig. 6D and Table S3). These results showed that pBMSC-EXOs can alleviate the cartilage damage induced by OA.

## Discussion

In the study, in addition to an immunophenotype similar, pBMSCs isolated from the specimens surgically excised from of children with polydactyly exhibited a greater ability than the BMSCs to proliferate and differentiate into chondrocytes. Furthermore, pBMSC-derived exosomes not only promoted the proliferation and migration of chondrocytes in vitro but also reduced the degree of joint injury in the OA model mice.

As pluripotent stem cells, BMSCs have played important roles in repairing and mitigating tissue damage in a variety of tissue damage models^31–33^. However, when articular cartilage is injured, spontaneous repair is very difficult because the perichondrium covering the articular cartilage surface is absent, bone progenitor cells are lost and the synthesis capacity of the chondrocytes adjacent to the injured site is limited^34^. Currently, BMSCs, adipose tissue-derived mesenchymal stem cells^35^, synovial mesenchymal stem cells^36^, and embryonic mesenchymal stem cells^37^ can be used as seed cells for tissue-engineered cartilage repair. Here, we demonstrated that pBMSCs have a great ability to differentiate into chondrocytes. The differentiation of stem cells into chondrocytes is a complex process regulated by multiple genes. Sox9 is an important early transcription factor for chondrogenesis and chondrocyte differentiation, and it is a sensitive factor in articular cartilage tissue engineering, which plays an important role in chondrogenesis^38,39^. Collagen type II (COL2A1), as a downstream target protein Sox9, is an important component of the cartilage matrix and is thought to be the basis of structural strength against outside pressure^40^. Similarly, aggrecan (Acan) is one of the main components of the ECM of articular cartilage and is necessary to maintain the normal shape of cartilage tissue^41^. In our study, we found that, compared with the levels in the BMSCs, the expression levels of COL2A1 and Acan were higher in the pBMSCs 14 days after chondrogenic differentiation, findings that confirm the predominance of the chondrogenic differentiation ability of pBMSCs.

In recent years, increasing evidence has suggested that stem cells and precursor cells may play important roles in promoting tissue regeneration by activating surrounding cells through paracrine mechanisms^42,43^. Most cells produce exosomes and play crucial roles in intercellular communication^44^. Moreover, exosomes have been shown to have biological functions similar to that of the cells from which they were derived, and direct use of these nanoparticles has no obvious adverse effects, such as immune rejection or tumorigenicity^45,46^. In addition, exosomes can also promote the proliferation of chondrocytes in vitro^28^. In this study, we used polydactyly tissue-derived BMSCs as “factories” to produce exosomes and found that pBMSC-EXOs enhanced the proliferation and migration of chondrocytes in vitro. However, pBMSC-EXOs did not affect the proliferation or migration of the FLSs from the OA patients, although FLSs are the main cells that mediate joint damage by secreting metalloproteinases. Previous studies have demonstrated that FLSs can induce osteoclastogenesis to promote bone absorption or release chemokines to recruit white blood cells to the joint, stimulating angiogenesis, which plays an important role in the initial phase of synovitis^47^. These results indicate that pBMSC-EXOs can promote the proliferation and migration of chondrocytes in vitro without causing adverse effects induced by the abnormal proliferation of synovial fibroblasts. In addition, we also confirmed that pBMSCs-EXOs led to a positive remission effect in the OA model, which was consistent with the previous protective effect shown with other types of stem cell-derived exosomes on OA models.

A variety of key signals, such as those from WNT, BMP, FGF, HH, etc. were involved in the process of chondrocyte induction and differentiation^48^. For example, WNT5A and WNT11 play important regulatory roles in the chondrogenic differentiation of MSCs^49^. In addition, BMP2 or BMP4 can promote the differentiation of human embryonic stem cells into chondrocytes, leading to increased expression of chondrogenic genes Sox5, ACAN and COL2A1^50^. Similarly, in this study, we found that BMP4 was highly expressed in the pBMSCs. Moreover, the pBMSCs transfected with BMP4 siRNAs were rarely able to differentiate into chondrocytes or promote the proliferation of chondrocytes in vitro. Therefore, BMP4 signaling plays an important role in the differentiation and proliferation of pBMSCs to chondrocytes in vitro.

In conclusion, pBMSC-EXOs promote not only the proliferation and migration of chondrocytes in vitro but also cartilage repair and delayed progression of OA in vivo. Therefore, pBMSC-EXOs are expected not only to resolve the bottleneck of low chondrocyte regeneration and poor repair capacity but also to show great potential and good prospects for transformation and application in the treatment of injuries or defects of articular cartilage.

## Supplementary information

supplementary material files

Figure S1

Figure S2

Figure S3

Figure S4

Figure S5

Figure S6
