# Supplementary material for: BMSC-derived exosomes from congenital polydactyly tissue alleviate osteoarthritis by promoting chondrocyte proliferation
Source: Cell Death Discov. 2020 Dec 10;6:142. doi: 10.1038/s41420-020-00374-z (PMC7730395; doi:10.1038/s41420-020-00374-z)
Supplement: Supplementary file 1 — supplementary material files [file 41420_2020_374_MOESM1_ESM.docx]

**Supplementary materials**

**1.** **Supplementary Materials and Methods**

**1.1 Cell transfection**

Human BMP4 si480, si1318, si1494 and the respective control siRNA (siNC) were purchased from GenePharma (Shanghai, China). pBMSCs were transfected with siRNA reagents using DharmaFECT 1 Transfection Reagent (Dharmacon) according to the manufacturer’s instructions. Transfection efficiency was determined by qRT-PCR and western blotting.

**1.2 Western blot analysis**

Protein was extracted from the pBMSCs using SDS buffer, resolved by SDS-polyacrylamide gels and then transferred to PVDF membranes. Primary antibodies against BMP4 (1:100, Abcam) and GAPDH (1:10000, MultiSciences) were used. Horseradish peroxidase (HRP)-conjugated secondary antibody (1:10000, MultiSciences) was used, and the result was visualized by an enhanced Pierce ECL western blotting substrate kit (Thermo Scientific/Pierce).

**1.3 Isolation and identification of Microvesicles (MVs)**

In order to isolate MVs from cell culture supernatants, we cultivated cells at 80% confluency for 48 h in culture medium supplemented with vesicle-depleted FBS, and then collected the supernatant. The supernatants were centrifuged for 5 min at 750 x g, 4 °C to deplete residual floating cells, and transferred into a new 15 mL tube and centrifuged again for 30 min at 2,000 x g, 4 °C to pellet larger cell debris. After that, the supernatants were centrifuged twice for 35 min at 14,000 x g, 4 °C. Finally, resuspend the MV pellet in 50 - 500 μL PBS, depending on the size of the pellet. In order to discriminate MVs from EXOs, the protein Tubulin can be used which should mainly be present on MVs.

**1.4 Exosome activity test**

Use PKH26 dye to interact with exosomes. According to the kit instructions, take 100μL of pBMSCs-derived exosome suspension and resuspend in Diluent C solution. Add another 1μl of PKH26 dye to 250μl Diluent C solution, and quickly mix after 5 minutes incubation at room temperature, an equal volume of fetal calf serum was added to stop the reaction. The supernatants were centrifuged twice for 2 h at 10,0000 x g, 4 °C, to obtain PKH26-labeled exosomes, then it was added to chondrocytes cultured in vitro, and the interaction between PKH26-labeled exosomes and cultured chondrocytes was observed at different time points under a fluorescence microscope.

**1.5 Fibroblast-like synoviocyte culture**

OA synovial tissue was obtained by surgical resection. Then, we removed the attached yellow adipose tissue and blood clots in the synovium with a surgical blade. Next, the whole synovium was cut into a tissue block of 0.5 cm^3^. Next, the tissue block was completely cut with an ophthalmic scissors until the meat was minced. The tissue block continued to be digested with 0.1% type I collagenase at 37°C for more than 4 h, at which time a single cell was isolated. Finally, fibroblast-like synoviocytes were collected and cultured with DMEM/F12 containing 10% FBS. All cells were used between passage 3 and 5. The use of FLSs was approved by the Ethics Committee of Soochow University (approval No. SUDA20200707H01).

**1.6 Immunofluorescence**

The synovial fibroblasts were incubated overnight with a primary antibody against sheep anti-human CD90 (1:20, Abcam), sheep anti-human podoplanin (1:20, Cell Signaling Technology) and sheep anti-human α-FAP (1:20, Cell Signaling Technology) at 4°C. Then, the cells were washed with PBS and incubated with HRP-conjugated secondary antibodies (Life Technologies, Duren, DE) for 30 min at room temperature. Furthermore, after washing, the cells continued to incubated with a TSATM system (Cyanine 3 System, PerkinElmer, MA, USA) for 10 min at room temperature. Finally, images were captured by fluorescence microscopy (Nikon Eclipse Ni, Tokyo, Japan) after the nuclei were stained with DAPI (SouthernBiotech, AL, USA).

**1.7 Flow cytometry**

For cell surface marker analysis, synovial fibroblasts were resuspended in PBS containing 0.5% FBS and stained with fluorescent-conjugated antibodies against CD90 (BD), podoplanin (Invitrogen), and hFAP (Minneapolis) for 30 min at 4°C. Specimens were subsequently analyzed by a Gallios flow cytometer and Kaluza software (Beckman Coulter).

**1.8 Wound-healing assay**

Before the scratch was made for the wound-healing assay, the synovial fibroblasts were grown to 90%-100% confluence. Then, the cell monolayers were scratched and washed with PBS. Finally, images of wound healing were captured by a microscope (Nikon Eclipse Ti, Tokyo, Japan) connected to a Nikon camera using NIS-Elements software at different time points (24 h and 72 h).

**2. Supplementary Figure legend**

**Figure S1. The expression of BMP4 after siRNA interference.** The chondrocytes were transduced with siRNA against BMP4, and then, the transfection efficiency was determined by qRT-PCR **(A)** and western blotting **(B)**.

**Figure S2. Effects of the pBMSC-EXOs and pBMSC-MVs on the migration and proliferation of chondrocytes. (A)** Western blot analysis of the EXOs-specific protein (CD9, CD63), and MVs-specific protein (Tubulin). **(B)** Wound-healing assays.

**Figure S3. Exosomes tracking test.** According to the kit instructions, exosomes were labeled with PKH26 dye. After that, we added PKH26-labeled exosomes to cultured Chondrocytes to observe the whole process of PKH26-labeled exosomes been phagocytized by Chondrocytes at different time points under a microscope.

**Figure S4. The expression of BMP4 after siRNA interference.** The chondrocytes were transduced with siRNA against BMP4, and then analyzed the expression of BMP4 in exosome from pBMSCs transfected with BMP4 siRNAs.

**Figure S5. The morphological and biological characteristics of the FLSs. (A)** The morphology of FLSs from two OA patients; scale bars, 100 μm**. (B)** Representative images of immunofluorescence for CD90, podoplanin, α-FAP in FLSs; scale bars, 100 μm. **(C)** FLSs were positive for CD90, podoplanin, and hFAP determined by flow cytometry.

**Figure S6. The migration ability of the FLSs under pBMSC-EXOs treatment.** FLS cultures were scratched, and then, images of the wound healing with or without pBMSC-EXOs treatment were captured with a microscope. Scale bars, 500 μm.

**3. Supplementary tables**

**Table S1.** **qRT-PCR primers**

| **Gene** | **Forward primer (5’- 3’)** | | **Reverse primer (5’- 3’)** |
| --- | --- | --- | --- |
| Acan | | CCCCTGCTATTTCATCGACCC | GACACACGGCTCCACTTGAT |
| Sox9 | | AGCGAACGCACATCAAGAC | CTGTAGGCGATCTGTTGGGG |
| CoL2A1 | | TGGACGCCATGAAGGTTTTCT | TGGGAGCCAGATTGTCATCTC |
| Foxc2 | | CCTCCTGGTATCTCAACCACA | GAGGGTCGAGTTCTCAATCCC |
| BMP4 | | ATGATTCCTGGTAACCGAATGC | CCCCGTCTCAGGTATCAAACT |
| CTNNB1 | | AGCTTCCAGACACGCTATCAT | CGGTACAACGAGCTGTTTCTAC |
| c-Myc | | GTCAAGAGGCGAACACACAAC | TTGGACGGACAGGATGTATGC |
| Actin | | CATGTACGTTGCTATCCAGGC | CTCCTTAATGTCACGCACGAT |

**Table S2. siRNA targeting sequences**

| **Sequences** |
| --- |
| **siRNA sense(5’→3’) antisense（5’→3’）** |
| siNC UUCUCCGAACGUGUCACGUTT ACGUGACACGUUCGGAGAATT |
| si-480 GAGCCAUGCUAGUUUGAUATT UAUCAAACUAGCAUGGCUCTT |
| si-1318 GCCAGGAAGAAGAAUAAGATT UCUUAUUCUUCUUCCUGGCTT |
| si-1494 CCUGGUCAAUUCUGUCAAUTT AUUGACAGAAUUGACCAGGTT |

**Table S3. The** **OARSI score in four groups**

| **Group** | **OARSI score** |
| --- | --- |
| Con | 0±1 |
| OA | 12.33±1.15 |
| BMSC-EXOs | 5.0±1**^/##^ |
| pBMSC-EXOs | 3.0±1**^/##/△^ |

***p* < 0.01, BMSC-EXOs and pBMSC-EXOs versus Con group; ^##^*p* < 0.01, BMSC-EXOs and pBMSC-EXOs versus OA group；^△^*P*<0.05, pBMSC-EXOs versus BMSC-EXOs group.
