# Supplementary figures and images for: BMSC-derived exosomes from congenital polydactyly tissue alleviate osteoarthritis by promoting chondrocyte proliferation
Source: Cell Death Discov. 2020 Dec 10;6:142. doi: 10.1038/s41420-020-00374-z (PMC7730395; doi:10.1038/s41420-020-00374-z)

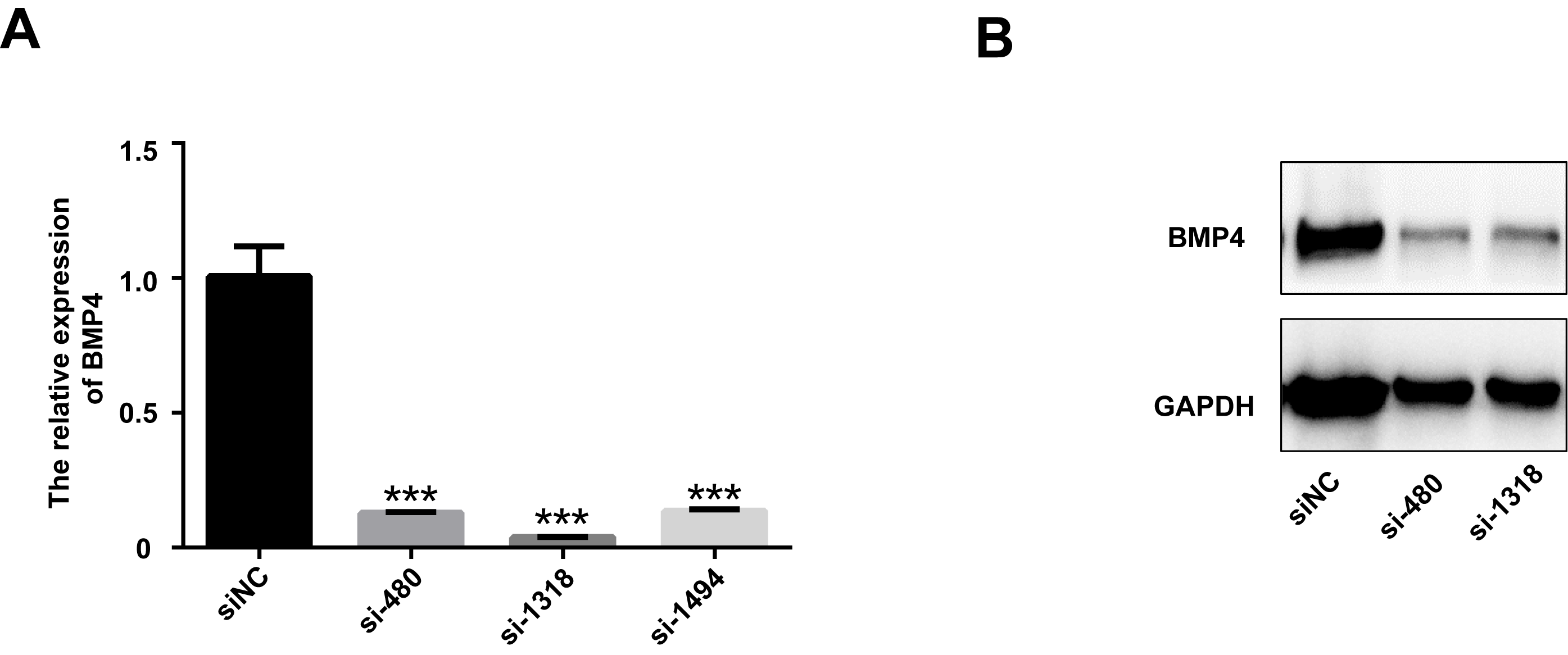

Supplement: Supplementary file 2 — Figure S1 [file 41420_2020_374_MOESM2_ESM.tif]

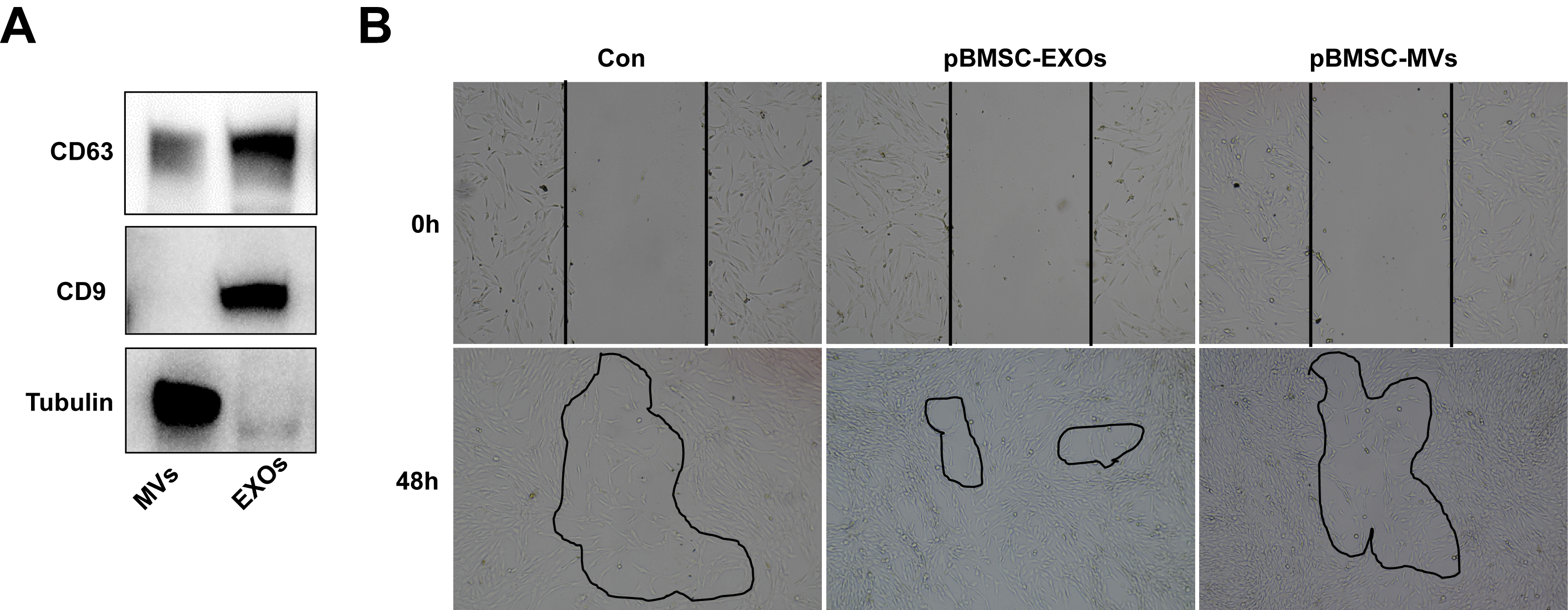

Supplement: Supplementary file 3 — Figure S2 [file 41420_2020_374_MOESM3_ESM.tif]

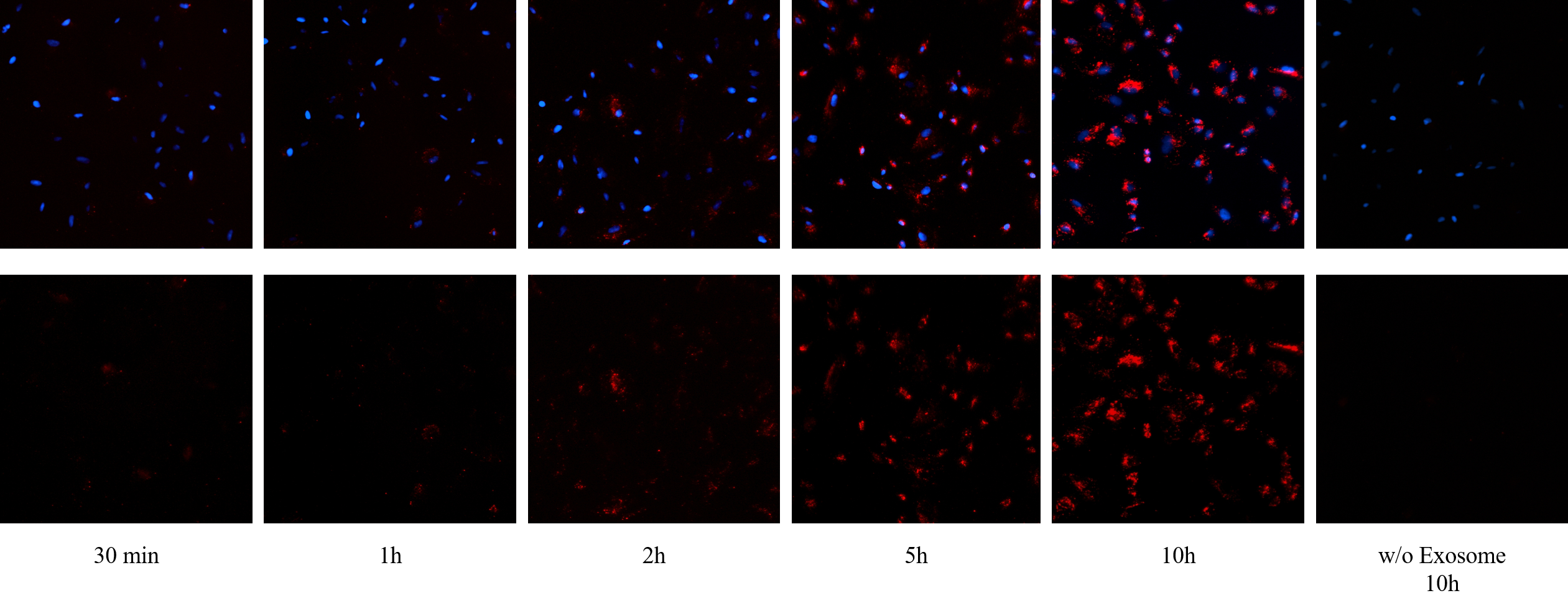

Supplement: Supplementary file 4 — Figure S3 [file 41420_2020_374_MOESM4_ESM.tif]

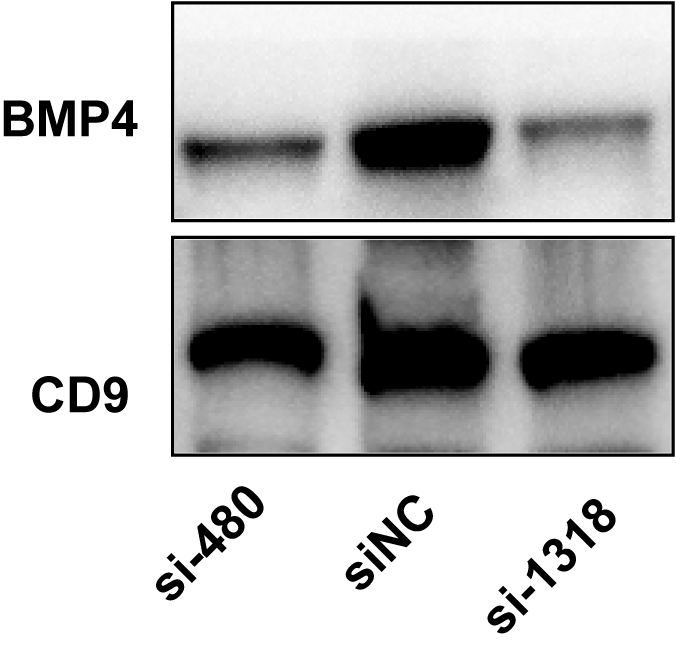

Supplement: Supplementary file 5 — Figure S4 [file 41420_2020_374_MOESM5_ESM.tif]

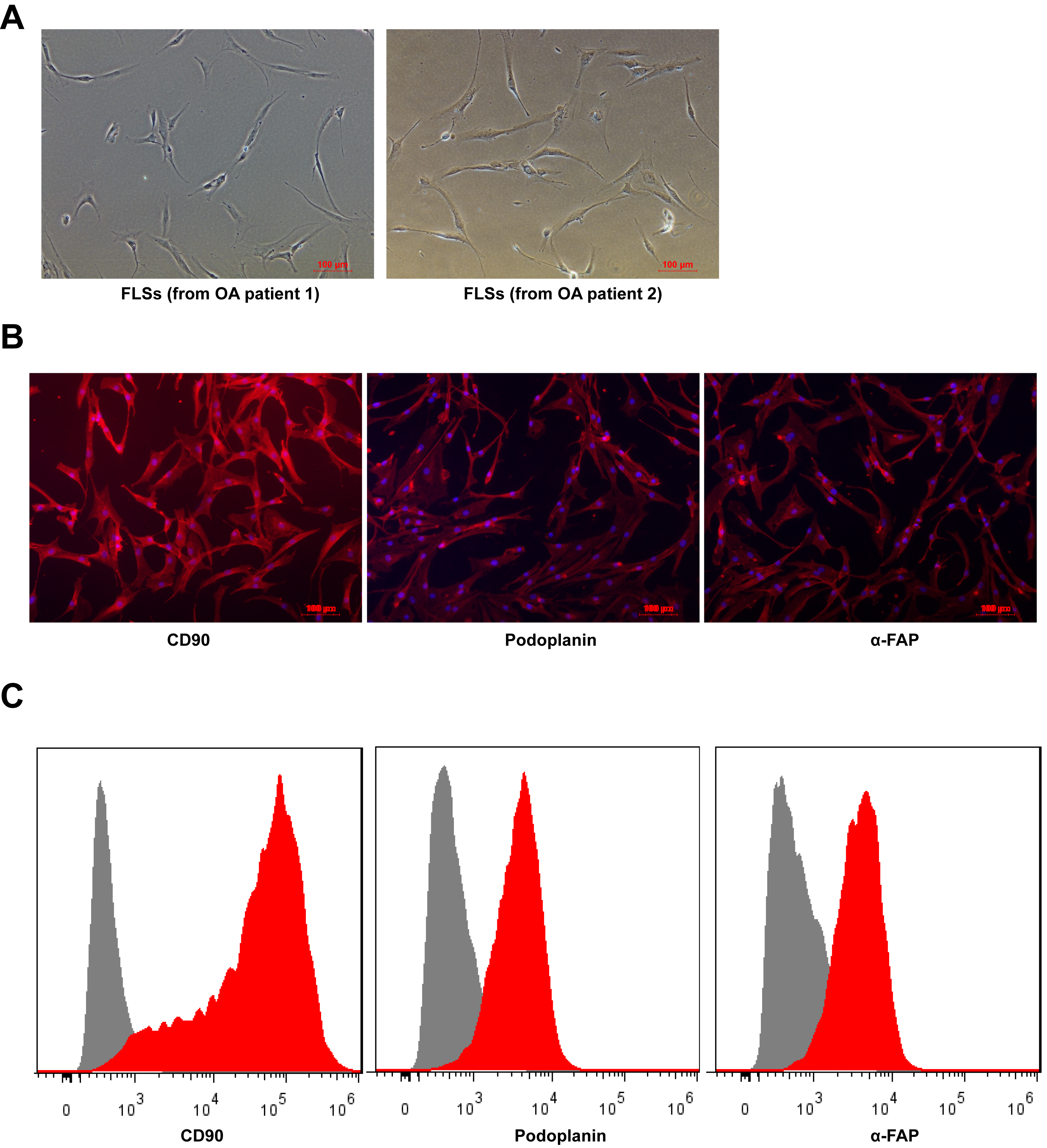

Supplement: Supplementary file 6 — Figure S5 [file 41420_2020_374_MOESM6_ESM.tif]

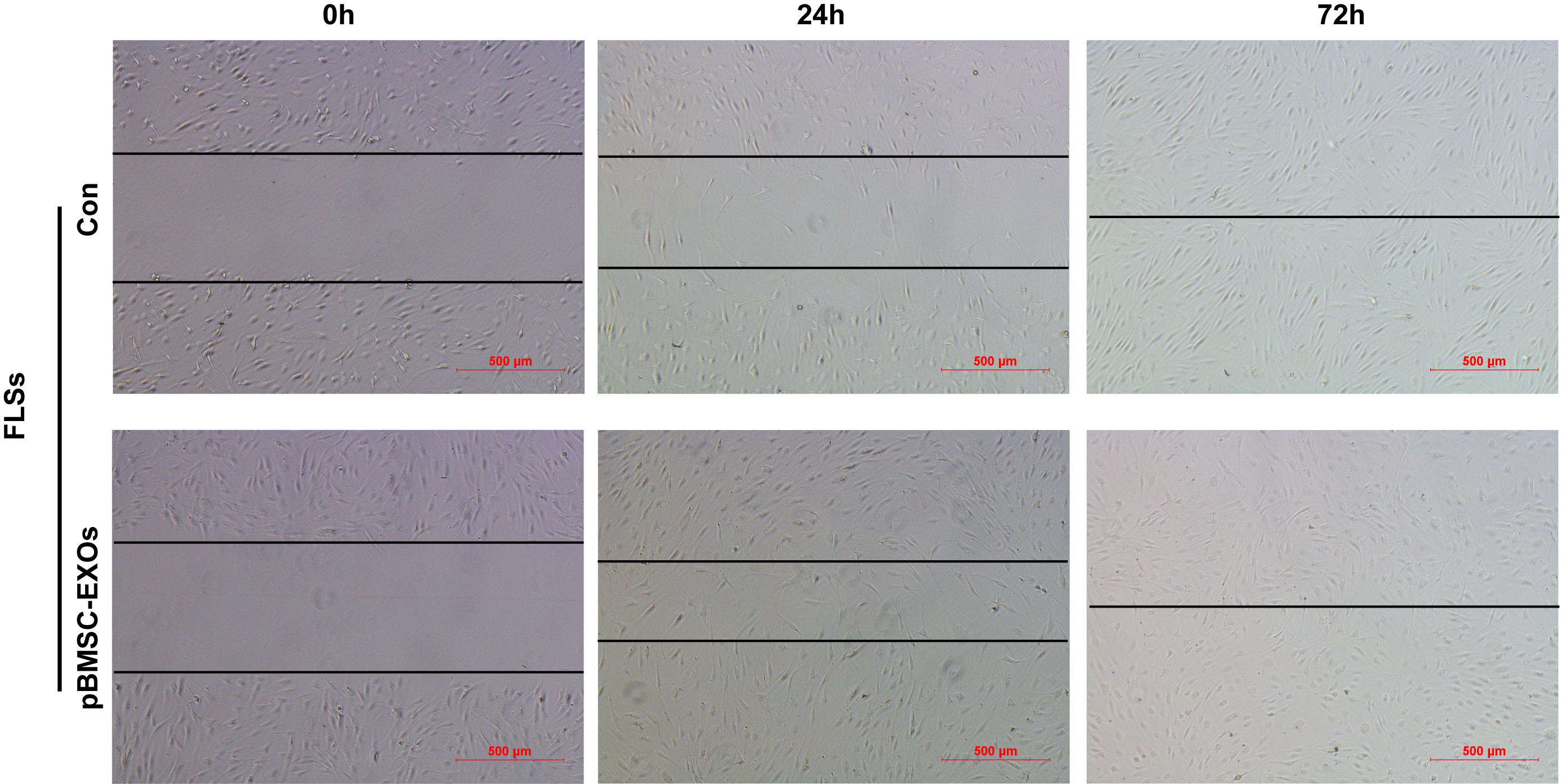

Supplement: Supplementary file 7 — Figure S6 [file 41420_2020_374_MOESM7_ESM.tif]
